# Supplementary figures and images for: Whole cell reconstructions of Leishmania mexicana through the cell cycle
Source: PLoS Pathog. 2024 Feb 28;20(2):e1012054. doi: 10.1371/journal.ppat.1012054 (PMC10927142; doi:10.1371/journal.ppat.1012054)

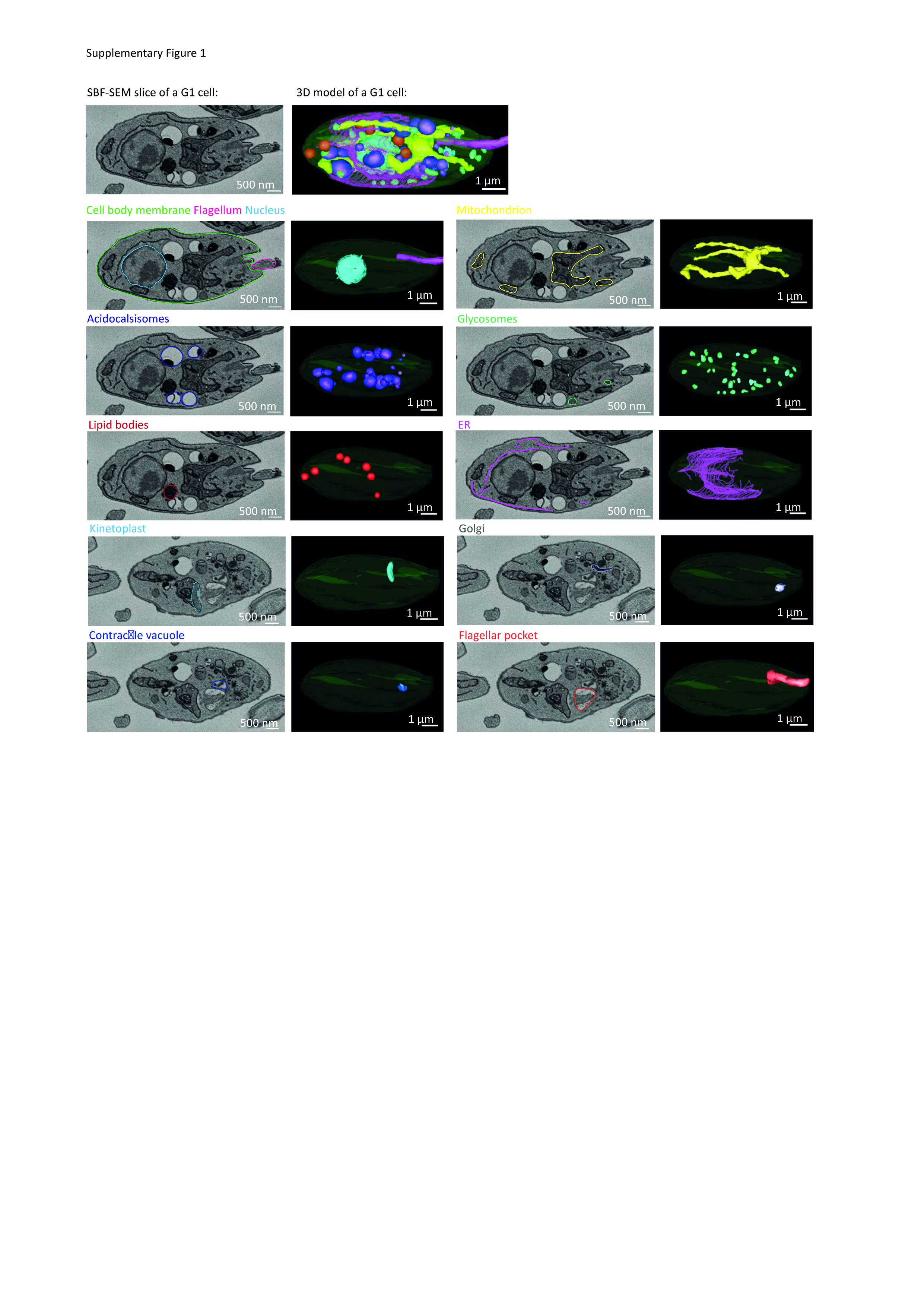

Supplement: S1 Fig — Each organelle was 3D reconstructed in a separate colour as defined in the key. (TIF) [file ppat.1012054.s004.tif]

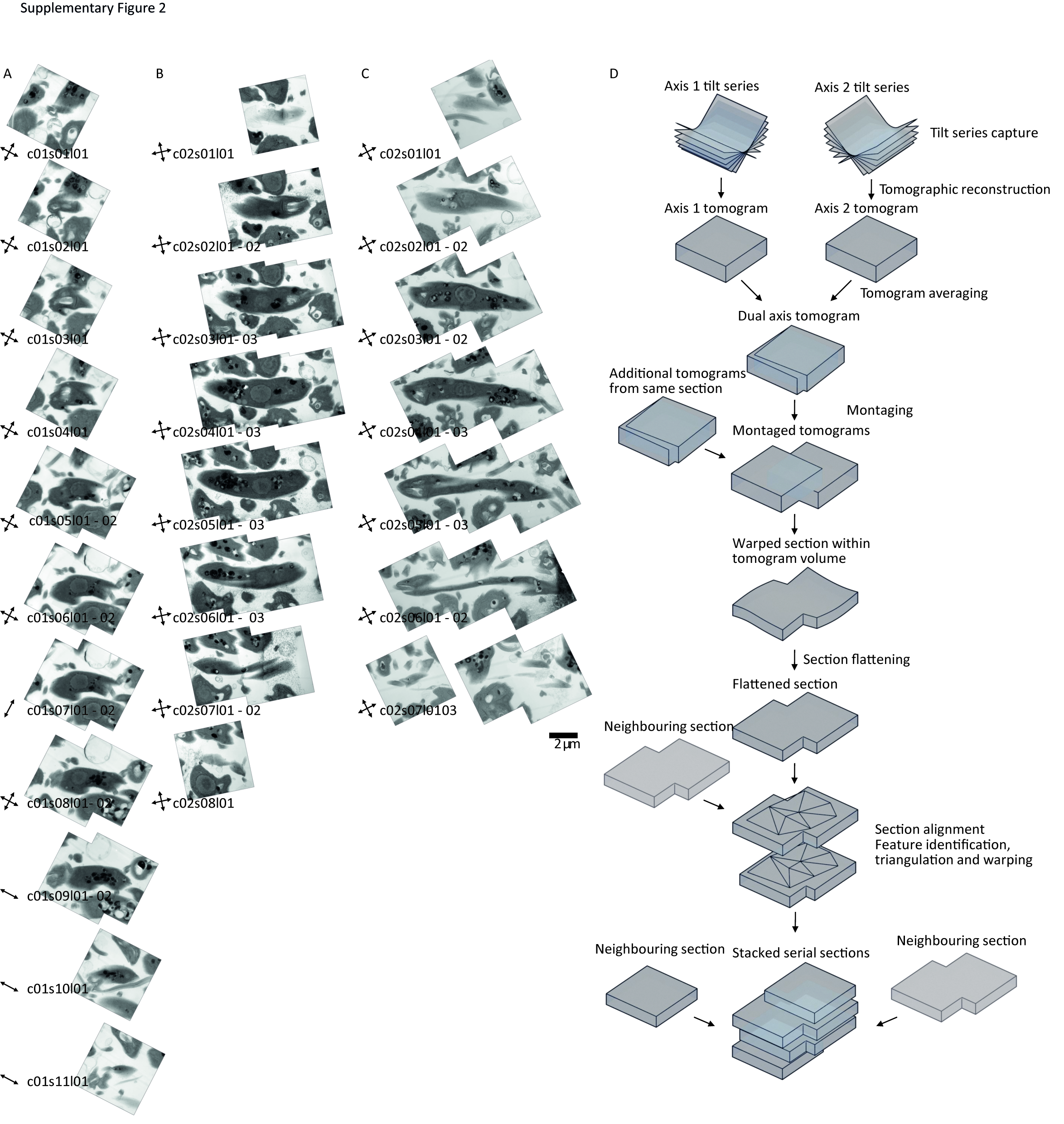

Supplement: S2 Fig — Fields of view on the serial sections for (A) the early G1 (16 tomograms, 11 serial sections), (B) the mid-G1 (18 tomograms, 8 serial sections) and (C) the S phase cells (17 tomograms, 7 serial sections). In each, sections are named with c indicating cell number, s indicating section number and l indicating the range of field of view location numbers–with each location being a tomogram. The arrows indicate the approximate tilt axes, with one arrow for sections captured with a single tilt axis, and the section name in bold is the centre section to which the other sections were aligned. A small region in c01s10l01 in which microtubule tracing was not possible and thus ends not mapped is circled, due to microtubule orientation in the single axis tomogram. (D) Summary of the strategy for accurate reconstruction of whole cells by tomography. (TIF) [file ppat.1012054.s005.tif]
