# Supplementary material for: Whole cell reconstructions of Leishmania mexicana through the cell cycle
Source: PLoS Pathog. 2024 Feb 28;20(2):e1012054. doi: 10.1371/journal.ppat.1012054 (PMC10927142; doi:10.1371/journal.ppat.1012054)
Supplement: Attachment — Submitted filename: Response to reviewers PLoS Pathogens.docx [file ppat.1012054.s007.docx]

Editor Comments

• Reviewer 1. Please make clear how new flagella were distinguished from old flagella (Figs. 1B and 2C). Is the distribution (Fig. 1E) consistent with prior studies? Please provide quantification (Fig. 2) confirming that the NF always forms to the right of the OF.

• Reviewer 2. Please discuss any ideas you may have about how organelles maintain their positions and then move when cells progress toward cytokinesis. Please include the two references mentioned by this reviewer by Akiyoshi regarding MAD2 and Marcelo Santos Da Silva, 2013.

• Reviewer 3. Please address this reviewer’s specific comments regarding Figs. 1E, 5I, and 5G-H.

If all of relevant points are addressed, I hope to be able to make a final decision without sending the manuscript back for a second round of review.

We have addressed the comments made by the reviewers through changes to the text and figures, and have responded in detail to them, please see below.

Reviewer Comments (if any, and for reference):

Reviewer's Responses to Questions

**Part I - Summary**
Please use this section to discuss strengths/weaknesses of study, novelty/significance, general execution and scholarship.

Reviewer #1: In this study, Hair et al used volume electron microscopy approaches to produce high resolution three-dimensional reconstructions of seven cell cycle stages of Leishmania mexicana promastigotes. It is an impressive collection of qualitative and quantitative analyses which should be valuable for the trypanosomatid cell biology community. While most results are consistent with previous studies on L. mexicana or other trypanosomatids, they do yield much better resolution. There is some new information including the dynamics of new flagellum formation, contractile vacuole, and nuclear pores which could lead to mechanistic and hypothesis-driven research in the future.

We thank the reviewer for their positive comments about our analyses of the Leishmania cell cycle.

Reviewer #2: The manuscript by Hair et al, uses state of the art scanning electron microscopy and 3D tomography reconstructions to analyze the organelle distribution and morphological changes of Leishmania mexicana promastigotes during cell division. The authors present a detail analysis of the patterns observed in asynchronous cell culture as well as promastigotes collected from the stomodeal valve of infected sand flies.
They elegantly show discrete stages during cell division and quantify the abundance and volume of organelles. Their data supports a highly coordinated control for organelle replication and segregation, and it has previously shown by other techniques with less resolution. The data collection and analysis is rigorous and presented in a clear way.
This work reinforces the idea of molecular mechanisms that tightly control the progression of the cell cycle in Leishmania, still to be elucidated. A question that I wish the authors would address is, based on their observations, how do they think the organelles maintaining their position and eventually moving when the cells change shape and progress toward cytokinesis. Have they observed association with cytosolic microtubules or other cytoskeletal elements? What triggers the duplication of organelles at such discrete time points? Incorporating some comments about these points in the Discussion would be a valuable addition to the manuscript, that, otherwise is outstanding.

Again, we thank the reviewer for their positive comments. We agree with the reviewer that cell cycle progression in *Leishmania* is tightly controlled and have included an additional section in the discussion about the movements of various organelles that would be required for their segregation. We have not observed any association between organelles and cytoskeletal elements other than the known flagellum, flagellar pocket, basal body, kinetoplast, and mitochondrion axis and the connection of the ER to the sub-pellicular microtubules but we cannot exclude additional connections existing. In terms of the molecular triggers little is known in *Leishmania* and so we have relied on parallels to *Trypanosoma brucei*. In 2020, Akyisohi in his preprint identified a role for MAD2 in the initiation of the cell cycle and we have now included reference to additional work from the Horn group who used genome-wide RNAi analysis to identify proteins important for cell cycle progression, including CRK7.

Reviewer #3: In this work, Hair and colleagues have employed SBF-SEM and tomographic reconstructions to to study the mechanism of cell division in promastigote Leishmania. The high information content and resolution of their datasets have allowed them to establish a set of cell division intermediates that identify the order of assembly of many key cellular components, such as the flagellum, FP, secretory pathway, and DNA-containing compartments. This work has led to confirmation of the order of events that had previously been seen using light microscopy, such as the timing of new flagellum growth and copy number of the contractile vacuole and Golgi. It also provides tantalizing glimpses into previously unknown phenomena, such as the association of the ER with non-MtQ subpellicular microtubules and the depletion of nuclear pores from the nuclear bridge. The fact that the cleavage furrow appears to ingress from both sides of the cell body and produces indentations that persist after the completion of cytokinesis is also a very interesting discovery.

We welcome such positive comments that highlight the significance of our work.

**Part II – Major Issues: Key Experiments Required for Acceptance**
Please use this section to detail the key new experiments or modifications of existing experiments that should be absolutely required to validate study conclusions.

Reviewer #1: The authors state that a total of 40 log phase promastigotes whose cell body and flagella lay within the imaged dataset were analyzed. I understand that flagella may be detached during processing but how often does that happen? In other words, how representative are those 40 cells amongst the seven stages?

In our hands we have not experienced flagella detachment during EM processing. There are in fact 41 cells in our dataset and we have corrected this throughout. The 41 cells we examined in detail consisted of 6 in Stage 1, 9 in Stage 2, 4 in Stage 3, 6 in Stage 4, 6 in Stage 5, 6 in Stage 6, and 4 in Stage 7. We therefore have a relatively even spread across the different cell cycle stages we examined. From our ergodic analysis in Figure 1E based on 500 random cells in the SBF-SEM we show that the first 4 Stages cover 0.8 u of the cell cycle and therefore we have an over-representation of the final stages of the cell cycle (Stages 5-7, 16 out of 41). However, these later stages contain important morphological changes for cell division which is a key focus of this work. We have included the number of cells we examined for each stage in the figure legend of Figure 1 and on the graphs in Figure 1, so people can more readily see this information.

Reviewer #2: (No Response)

Reviewer #3: Overall, the quality of the work in the manuscript is excellent. The collection of imaging provides is a valuable resource to the field and provides a series of observations that could be the foundation for subsequent work. I will say that several of the observations tend to lack detail or context- such as the cap structure at the end of the flagellum and the ER-microtubule association. The lack of perturbations, either chemical or via protein knockout, means that we are looking at a purely observational body of work. I think the scope of the work and degree of quantitation do mitigate this issue.

We thank the reviewer for highlighting the quality of our work and the fact it will be an important resource for the field. We have provided more context to the results about the cap structure at the distal tip of the flagellum and more details to the results about the ER-microtubule association.

**Part III – Minor Issues: Editorial and Data Presentation Modifications**
Please use this section for editorial suggestions as well as relatively minor modifications of existing data that would enhance clarity.

Reviewer #1: The last sentence of the abstract “Our insights into the cell cycle mechanics of Leishmania will be invaluable for future molecular cell biology analyses of these important parasites.” Seems a bit too strong and self-serving and needs to be toned down.

We have reworded this sentence, toning down the conclusion and we now state that our insights will support future cell biology analyses.

Fig. 1B and Fig. 2C: It is not clear how new flagella (NF) are distinguished from the old flagella. Do NF not have PFR? If so, when is the PFR made for the NF? The information should be referenced if available.

In *Leishmania*, the flagellum is not disassembled during the cell cycle and when a cell begins to assemble a new flagellum, the old flagellum is always longer than the new flagellum throughout the cell cycle. This enables us to unequivocally distinguish between the old and new flagellum in all cell cycle stages. We have clarified in the text that the old flagellum is longer than the new flagellum. The new flagellum does have a PFR and the PFR is first observed as the new flagellum extends out of the cell body as shown in Stage 3.

Fig. 1E: no error bars were included. Is the distribution consistent with prior studies on promastigote cell cycle using other approaches (e.g., fluorescence microscopy)?

This analysis was based on 500 random cells in the SBF-SEM run we analysed. We only made this count once, so we are unable to include error bars. However, our analysis is comparable with previous analyses. By various approaches using fluorescence microscopy S-phase was shown to begin at 0.35-0.4 u and post S-phase 0.78-0.86 u (Wheeler et al., 2011), and here we found an increase in nuclear volume suggestive of S-phase to occur at 0.3 u and S-phase to be complete by 0.8 u, therefore in line with previous work. In the discussion, we have a section where we talk about the variability that has been seen in the progression of the *Leishmania* cell cycle and how our data fits with these references.

Line 148: The word “dividing” is misspelled.

Thank you for spotting this, we have changed it.

Fig. 2: Some kind of quantitation is required to establish the conclusion that NF always forms to the right of OF.

We investigated the positioning of the new flagellum relative to the old flagellum in 25 cells. In every cell we examined the new flagellum was in a consistent position relative to the old flagellum. We have included this number in the text now.

Fig. 4. “Nasecent” seems to a misspelling of nascent.

Thank you for spotting this, we have changed it.

Fig. 6E-H: the labelling for sand fly and cultured promastigotes seems to be flipped (the text states sand fly parasites are smaller than culture promastigotes).

Thank you for spotting this, we have changed it.

Reviewer #2: Minor corrections/questions:
- Why did you decide to look at the cells in asynchronous culture vs synchronous? Analyzing synchronized cells could provide clearer evidence on when does the new flagellum assembly starts (lines 427-430).

In culture *Leishmania* parasites grow in an asynchronous manner and we were able to use ergodic principles to allow us to estimate the timing of specific cell cycle events. While the use of parasites with a synchronised cell cycle would have been informative to confirm our observations, the most appropriate approaches for cell cycle synchronisation are not yet available for *Leishmania.*

There are two main approaches that could be used to synchronise the cell cycle of Trypanosomes for example are cell cycle inhibitors and elutriation. Cell cycle inhibitors such as hydroxyurea, cause a block at a specific point in the cell cycle, in this case the G1/S phase transition. The inhibitor can then be removed releasing the block and the cells then proceed through the first cell cycle in synchrony. However, inhibitors such as these are associated with morphological changes to the cell and so we do not think they would be suitable for our morphology focussed analysis. Elutriation based approaches have been successfully used to synchronise cell cycle of the closely related parasite *T. brucei* but to the best of our knowledge this approach has not been used for *Leishmania*. Elutriation would likely provide a population of cells to define the timing of specific cell cycle events such as new flagellum assembly, but the development of this approach for *Leishmania* was not within the scope of this current work.

- The basal body is not represented in the reconstructions, even though is a key structure for the formation of the new flagellum. Could you explain why? Is it due to a technical limitation?

We agree with the reviewer that these are important structures. However, in SBF-SEM datasets it is difficult to identify and segment the basal body and associated pro-basal body reliably and therefore we decided against including them in our analysis.

- Lines 27-29: please, revise the grammar of the sentence "We showed that..."

We have modified this sentence.

- Replace Golgi Body in for Golgi, as all the figure labels indicate only Golgi.

We only refer to the Golgi in the text now.

- Line 148 replace (dividng) for (dividing)

Thank you for spotting this, we have changed it.

- In line 435 you cite the work of Akiyoshi regarding the possible control of the cell cycle initiation by MAD2. This is a preprint from 2020 that has not been peer-reviewed published. Are there other references supporting the role of this or other proteins in initiating cell cycle in trypanosomatids? This is a pending question that needs further study. It would be good to add more supporting literature.

The work concerning MAD2 has yet to be published but given it speaks directly to the initiation of the cell cycle we thought it was important to include here. A recent genome wide RNAi screen by David Horn and his group identified a number of proteins important for the G1/S-phase transition, including many glycolytic enzymes showing the importance of monitoring the cell’s metabolic status before initiating the cell cycle and the cell cycle associated kinase, CRK7. We have included this data in the discussion.

- The work by Marcelo Santos Da Silva, 2013 in Leishmania amazonensis is an important reference that should be mentioned in the manuscript (lines 436-439).

Thank you for your comment. We have included this reference in the manuscript.

Reviewer #3: Fig 1E- hard to see 6/7 on very dark grey background in the graph. I would recommend altering the color scheme.

Thank you for your comment, we have changed the colour scheme.

5I: this is a very interesting way to represent the subpellicular microtubules. While the seam in the first stage 1 cell is fairly clear, in the second cell it looks like there are couple other features that look similar to what is called the seam, along with regions in the Stage 2 cells. Is it possible to employ a statistical test to show that the MT length in the seam region is significantly different from the rest of the array?

We completely agree that the 'seam' is less convincing in the late G1 cell, and that some of the other features could arguably be interpreted as a seam instead - which is, of course, the key conclusion here: that the seam gets resolved by later cell cycle stages. We have carried out an additional analysis of the radial position of starts and ends of microtubules around the cell to provide a slightly more quantitative analysis. This shows a highly convincing single peak for radial position of microtubule ends in the first Stage 1 cell, multiple peaks for the second Stage 1 cell, and a uniform distribution for the Stage 2 cell. We have included the radial distribution of microtubule ends in the cell as part of Figure 5 to help illustrate this point. Statistically, the first and second Stage 1 cells deviate from a uniform radial distribution (Chi squared test vs. uniform distribution, p = 1.1e-12, 3.3e-6 and 0.31 respectively). However, we were unable to identify a suitable statistical test for if there are one or multiple peaks, and there is additional unaddressed complexity about where the microtubule ends are - close to the cell posterior or distant.

In Fig 5G-H, is is possible to use these numbers to estimate if the switch from stage 1 to stage 2 requires the production of more tubulin by the cells, or is the total net quantity of tubulin the same, with the depolymerized tubulin being used to extend the remaining microtubules? There would be some caveats to the measurement either way, but it would be very interesting if the net amount of tubulin in Stage 1 vs Stage 2 was similar.

In short, yes - you can estimate total tubulin quantity. The total microtubule length per cell is easily derived from the electron tomography data:

Early G1 - 771.63 µm

Late G1 - 844.10 µm

S phase - 936.19 µm

However, the better statistic for considering total sub-pellicular microtubule tubulin quantity is likely the cell surface area measure from the SBF-SEM dataset. All our observations confirmed the expected even spacing of sub-pellicular microtubules across the entire sub-pellicular array, and that the array indeed covers essentially the entire cell surface. As such, the cell surface area is, to a close approximation, proportional to quantity of sub-pellicular tubule tubulin. The relationship can be estimated from the 8 nm pitch of a microtubule dimer, 13 protofilaments, and ~20 nm separation of microtubules. This yields 1000 / 8 x 1000 / 20 x 13 = 81,250 tubulin dimers per square micron of cell surface, or 1000 / 20 = 50 µm of microtubule per square micron of cell surface. We have now included an additional sentence in the text that highlights that the cell surface area is proportional to total amount of microtubules/tubulin needed for the sub-pellicular array.
